# [(Methyl­carbamothio­yl)disulfan­yl]methyl *N*-methyl­carbamodithio­ate

**DOI:** 10.1107/S160053681003833X

**Published:** 2010-09-30

**Authors:** Hizbullah Khan, Muhammad Aziz, Christine Neuhausen, Ghulam Murtaza, Farkhanda Shaheen

**Affiliations:** a Department of Chemistry, Quaid-i-Azam University, Islamabad, Pakistan; b Department of Chemistry, University of Science and Technology, Mirpur AJK, Pakistan; c Laboratoire de Cristallographie, Ecole Polytechnique Fédérale de Lausanne, Switzerland

## Abstract

The title compound, C_5_H_10_N_2_S_5_, was unintentionally obtained as the product of an attempted synthesis of a methyl­carbamodithioic acid using methyl­amine and carbon disulfide. In the mol­ecule, two dithio­carbamate groups are bridged by a –CH_2_S– unit. The C—S—S—C torsion angle is −90.13 (11)°. The crystal structure is stabilized by N—H⋯S inter­actions between neighbouring mol­ecules. An intra­molecular N—H⋯S hydrogen bond also occurs.

## Related literature

For dithio­carbamate ligands, see: Cox *et al.* (1999 ▶); Liu & Bao (2007 ▶); Nair *et al.* (2002 ▶).
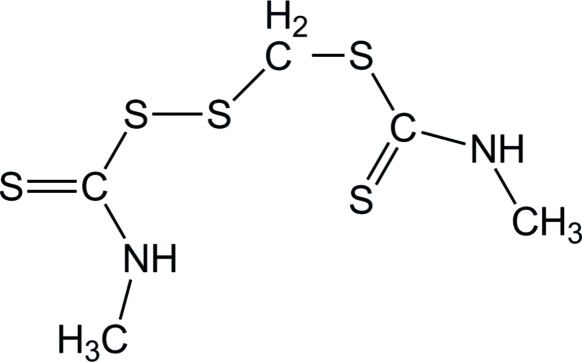


## Experimental

### 

#### Crystal data

C_5_H_10_N_2_S_5_
*M* *_r_* = 258.45
Triclinic, 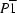

*a* = 7.188 (1) Å
*b* = 7.884 (2) Å
*c* = 10.219 (2) Å
α = 101.23 (3)°
β = 96.85 (3)°
γ = 102.74 (3)°
*V* = 546.0 (2) Å^3^
*Z* = 2
Mo *K*α radiation
μ = 1.01 mm^−1^
*T* = 293 K
0.30 × 0.11 × 0.10 mm

#### Data collection

Stoe IPDS diffractometer
4080 measured reflections
2077 independent reflections
1924 reflections with *I* > 2σ(*I*)
*R*_int_ = 0.101

#### Refinement

*R*[*F* ^2^ > 2σ(*F* ^2^)] = 0.040
*wR*(*F* ^2^) = 0.103
*S* = 1.15
2077 reflections
111 parameters
H-atom parameters constrained
Δρ_max_ = 0.53 e Å^−3^
Δρ_min_ = −0.37 e Å^−3^

### 

Data collection: *X-AREA* (Stoe & Cie, 2002 ▶); cell refinement: *X-RED32* (Stoe & Cie, 2002 ▶); data reduction: *X-RED32*; program(s) used to solve structure: *SHELXS97* (Sheldrick, 2008 ▶); program(s) used to refine structure: *SHELXL97* (Sheldrick, 2008 ▶); molecular graphics: *DIAMOND* (Brandenburg, 2007 ▶); software used to prepare material for publication: *enCIFer* (Allen *et al.*, 2004 ▶).

## Supplementary Material

Crystal structure: contains datablocks I, global. DOI: 10.1107/S160053681003833X/om2358sup1.cif

Structure factors: contains datablocks I. DOI: 10.1107/S160053681003833X/om2358Isup2.hkl

Additional supplementary materials:  crystallographic information; 3D view; checkCIF report

## Figures and Tables

**Table 1:** Hydrogen-bond geometry (Å, °)

*D*—H⋯*A*	*D*—H	H⋯*A*	*D*⋯*A*	*D*—H⋯*A*
N1—H1⋯S3	0.86	2.50	3.073 (2)	125
N1—H1⋯S3^i^	0.86	3.02	3.595 (2)	127
N2—H2⋯S1^ii^	0.86	2.67	3.515 (2)	168

Symmetry codes: (i) 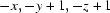; (ii) 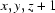.

**Fig. 1.**
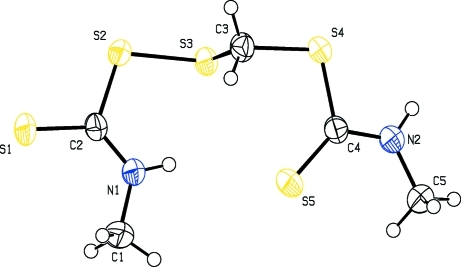
The molecular structure with atom labels and 50% probability displacement ellipsoids for non-H atoms.

## supplementary crystallographic information

### Comment

Sulfur-containing organic compounds like dithiocarbamates and xanthates have been used as exceleant metal complexing agents. They have applications as fungicides, pesticides, chelating agents for removal of heavy metal ions from toxic waste, precursors for metal-organic chemical vapour deposition (MOCVD) and synthesis of semi-conductor nanoparticles (Cox & Tiekink, *et al.*, 1999; Nair *et al.*, 2002.) Dithiocarbamates have also been used as protection groups in peptide synthesis, as linkers in solid phase organic synthesis and recently in the synthesis of ionic ligands (Liu *et al.*, 2007.) In the title compound (Fig. 1), the disulfide portion is substatially twisted, with C–S–S–C torsion angle of -90.13 (11)°. The molecular packing also features intra- and intermolecular N—H···S interactions (Table 1).

### Experimental

Distilled methylamine (3.00 g, 96.8 mmol) was added in purified methanol (30 ml) in a two neck flask (250 ml) and stirred for ten minutes at 273 K. Carbon disulfide 7.4 ml (117 mmol) was added drop by drop into the two neck flask containing methylamine and a colorless precipitate was formed at once. The stirring was continued for three hours to complete the reaction. The solvent was removed by vacuum distillation. The solid product was washed several times with methanol. The colorless product was purified by recrystallization from 1,1-dichloromethane/pet ether (8:2) V/V), to give fine crystals of the title compound with an overall yield of 85%.

### Refinement

All hydrogen atoms were initially located in a difference Fourier map. H atoms on C and N were refined with a riding model, C-H = 0.96 Å with U_iso(H)_ = 1.5U_eq_(C) for methyl groups, C-H = 0.97 Å with U_iso(H)_ = 1.2 U_eq_(C) for methylene groups, and N-H = 0.86 Å with U_iso(H)_ = 1.2 U_eq_(C).

### Crystal data

**Table:**

C_5_H_10_N_2_S_5_	*V* = 546.0 (2) Å^3^
*M**_r_* = 258.45	*Z* = 2
Triclinic, *P*1	*F*(000) = 268
*a* = 7.188 (1) Å	*D*_x_ = 1.572 Mg m^−^^3^
*b* = 7.884 (2) Å	Mo *K*α radiation, λ = 0.71073 Å
*c* = 10.219 (2) Å	µ = 1.01 mm^−^^1^
α = 101.23 (3)°	*T* = 293 K
β = 96.85 (3)°	Needle, yellow
γ = 102.74 (3)°	0.30 × 0.11 × 0.10 mm

### Data collection

**Table:**

Stoe IPDS diffractometer	1924 reflections with *I* > 2σ(*I*)
Radiation source: fine-focus sealed tube	*R*_int_ = 0.101
graphite	θ_max_ = 26.4°, θ_min_ = 4.1°
ω scans	*h* = −8→8
4080 measured reflections	*k* = −9→9
2077 independent reflections	*l* = −12→12

### Refinement

**Table:**

Refinement on *F*^2^	Primary atom site location: structure-invariant direct methods
Least-squares matrix: full	Secondary atom site location: difference Fourier map
*R*[*F*^2^ > 2σ(*F*^2^)] = 0.040	Hydrogen site location: inferred from neighbouring sites
*wR*(*F*^2^) = 0.103	H-atom parameters constrained
*S* = 1.15	*w* = 1/[σ^2^(*F*_o_^2^) + (0.0496*P*)^2^ + 0.0335*P*] where *P* = (*F*_o_^2^ + 2*F*_c_^2^)/3
2077 reflections	(Δ/σ)_max_ = 0.001
111 parameters	Δρ_max_ = 0.53 e Å^−^^3^
0 restraints	Δρ_min_ = −0.37 e Å^−^^3^

### Special details

**Table:**

Geometry. All e.s.d.'s (except the e.s.d. in the dihedral angle between two l.s. planes) are estimated using the full covariance matrix. The cell e.s.d.'s are taken into account individually in the estimation of e.s.d.'s in distances, angles and torsion angles; correlations between e.s.d.'s in cell parameters are only used when they are defined by crystal symmetry. An approximate (isotropic) treatment of cell e.s.d.'s is used for estimating e.s.d.'s involving l.s. planes.
Refinement. Refinement of *F*^2^ against ALL reflections. The weighted *R*-factor *wR* and goodness of fit *S* are based on *F*^2^, conventional *R*-factors *R* are based on *F*, with *F* set to zero for negative *F*^2^. The threshold expression of *F*^2^ > σ(*F*^2^) is used only for calculating *R*-factors(gt) *etc*. and is not relevant to the choice of reflections for refinement. *R*-factors based on *F*^2^ are statistically about twice as large as those based on *F*, and *R*- factors based on ALL data will be even larger.

### Fractional atomic coordinates and isotropic or equivalent isotropic displacement parameters (Å^2^)

**Table:**

	*x*	*y*	*z*	*U*_iso_*/*U*_eq_	
S1	0.24777 (9)	0.73176 (9)	0.11879 (5)	0.04736 (19)	
S2	0.39011 (7)	0.69015 (8)	0.38202 (5)	0.03952 (18)	
S3	0.28393 (7)	0.64232 (7)	0.55098 (5)	0.03611 (17)	
S4	0.22813 (8)	0.85040 (8)	0.80525 (5)	0.04221 (18)	
S5	−0.14480 (9)	0.82971 (9)	0.62019 (6)	0.04559 (19)	
N1	0.0187 (3)	0.6694 (2)	0.30088 (18)	0.0370 (4)	
H1	0.0090	0.6510	0.3803	0.044*	
N2	−0.1092 (3)	0.7742 (3)	0.86906 (19)	0.0440 (5)	
H2	−0.0358	0.7639	0.9384	0.053*	
C1	−0.1562 (3)	0.6703 (4)	0.2139 (3)	0.0485 (6)	
H1A	−0.1370	0.7799	0.1837	0.073*	
H1B	−0.2619	0.6604	0.2634	0.073*	
H1C	−0.1851	0.5713	0.1369	0.073*	
C2	0.1897 (3)	0.6948 (3)	0.26569 (19)	0.0333 (4)	
C3	0.3067 (3)	0.8671 (3)	0.6478 (2)	0.0390 (5)	
H3A	0.4402	0.9359	0.6636	0.047*	
H3B	0.2275	0.9267	0.5983	0.047*	
C4	−0.0280 (3)	0.8118 (3)	0.7646 (2)	0.0346 (4)	
C5	−0.3151 (3)	0.7494 (4)	0.8732 (3)	0.0488 (6)	
H5A	−0.3432	0.8638	0.8978	0.073*	
H5B	−0.3498	0.6812	0.9388	0.073*	
H5C	−0.3881	0.6867	0.7856	0.073*	

### Atomic displacement parameters (Å^2^)

**Table:**

	*U*^11^	*U*^22^	*U*^33^	*U*^12^	*U*^13^	*U*^23^
S1	0.0507 (4)	0.0674 (4)	0.0306 (3)	0.0158 (3)	0.0153 (2)	0.0206 (3)
S2	0.0326 (3)	0.0578 (4)	0.0331 (3)	0.0132 (2)	0.0107 (2)	0.0168 (3)
S3	0.0393 (3)	0.0427 (3)	0.0300 (3)	0.0103 (2)	0.0079 (2)	0.0157 (2)
S4	0.0366 (3)	0.0623 (4)	0.0270 (3)	0.0115 (3)	0.0030 (2)	0.0107 (3)
S5	0.0446 (3)	0.0627 (4)	0.0328 (3)	0.0181 (3)	0.0012 (2)	0.0168 (3)
N1	0.0357 (9)	0.0483 (10)	0.0304 (8)	0.0113 (8)	0.0081 (7)	0.0145 (8)
N2	0.0385 (10)	0.0645 (12)	0.0333 (9)	0.0154 (9)	0.0056 (8)	0.0187 (9)
C1	0.0379 (13)	0.0657 (15)	0.0452 (12)	0.0161 (11)	0.0032 (10)	0.0193 (12)
C2	0.0388 (11)	0.0358 (9)	0.0276 (9)	0.0105 (8)	0.0077 (8)	0.0104 (8)
C3	0.0413 (11)	0.0417 (11)	0.0331 (10)	0.0045 (9)	0.0093 (9)	0.0116 (9)
C4	0.0385 (11)	0.0382 (10)	0.0290 (9)	0.0136 (8)	0.0061 (8)	0.0073 (8)
C5	0.0395 (13)	0.0686 (16)	0.0417 (12)	0.0180 (12)	0.0101 (10)	0.0137 (12)

### Geometric parameters (Å, °)

**Table:**

S1—C2	1.6671 (18)	N2—C5	1.456 (3)
S2—C2	1.767 (2)	N2—H2	0.8600
S2—S3	2.0364 (8)	C1—H1A	0.9600
S3—C3	1.816 (2)	C1—H1B	0.9600
S4—C4	1.782 (2)	C1—H1C	0.9600
S4—C3	1.787 (2)	C3—H3A	0.9700
S5—C4	1.655 (2)	C3—H3B	0.9700
N1—C2	1.306 (3)	C5—H5A	0.9600
N1—C1	1.453 (3)	C5—H5B	0.9600
N1—H1	0.8600	C5—H5C	0.9600
N2—C4	1.328 (3)		
			
C2—S2—S3	105.99 (7)	S1—C2—S2	113.24 (12)
C3—S3—S2	101.85 (7)	S4—C3—S3	107.88 (10)
C4—S4—C3	103.25 (10)	S4—C3—H3A	110.1
C2—N1—C1	123.90 (17)	S3—C3—H3A	110.1
C2—N1—H1	118.0	S4—C3—H3B	110.1
C1—N1—H1	118.0	S3—C3—H3B	110.1
C4—N2—C5	123.93 (18)	H3A—C3—H3B	108.4
C4—N2—H2	118.0	N2—C4—S5	125.43 (17)
C5—N2—H2	118.0	N2—C4—S4	109.93 (14)
N1—C1—H1A	109.5	S5—C4—S4	124.59 (12)
N1—C1—H1B	109.5	N2—C5—H5A	109.5
H1A—C1—H1B	109.5	N2—C5—H5B	109.5
N1—C1—H1C	109.5	H5A—C5—H5B	109.5
H1A—C1—H1C	109.5	N2—C5—H5C	109.5
H1B—C1—H1C	109.5	H5A—C5—H5C	109.5
N1—C2—S1	127.70 (16)	H5B—C5—H5C	109.5
N1—C2—S2	119.06 (14)		
			
C2—S2—S3—C3	−90.13 (11)	S2—S3—C3—S4	−177.57 (9)
C1—N1—C2—S1	−0.4 (3)	C5—N2—C4—S5	−2.4 (3)
C1—N1—C2—S2	179.97 (18)	C5—N2—C4—S4	175.1 (2)
S3—S2—C2—N1	−0.01 (19)	C3—S4—C4—N2	172.23 (16)
S3—S2—C2—S1	−179.71 (9)	C3—S4—C4—S5	−10.31 (17)
C4—S4—C3—S3	−83.86 (14)		

### Hydrogen-bond geometry (Å, °)

**Table:**

*D*—H···*A*	*D*—H	H···*A*	*D*···*A*	*D*—H···*A*
N1—H1···S3	0.86	2.50	3.073 (2)	125
N1—H1···S3^i^	0.86	3.02	3.595 (2)	127
N2—H2···S1^ii^	0.86	2.67	3.515 (2)	168

Symmetry codes: (i) −*x*, −*y*+1, −*z*+1; (ii) *x*, *y*, *z*+1.
